# A Novel, Combined Endoscopic Technique to Reveal a Gastric Ulcer Underlying a Left Gastric Artery Pseudoaneurysm

**DOI:** 10.1002/jgh3.70132

**Published:** 2025-03-07

**Authors:** Jinye Liu, Abdulazeez Swaiti, Francesco Giorgino, Saeed Graham, Zarak Hassan Khan, Edward Barcelona, Karissa Lambert

**Affiliations:** ^1^ Department of Internal Medicine ECU Health Medical Center Greenville North Carolina USA; ^2^ Department of Gastroenterology ECU Health Medical Center Greenville North Carolina USA

**Keywords:** endoscopy, gastric ulcer, pancreatitis, pseudoaneurysm

## Abstract

Pseudoaneurysms, or false aneurysms, result from an arterial wall tear and can arise from trauma, infection, or inflammation. Common types include aortic, cardiac, and femoral pseudoaneurysms, while left gastric artery pseudoaneurysms (LGAP) are rare visceral occurrences, with only a handful of documented cases. LGAPs are often associated with recurrent pancreatitis and require prompt recognition and treatment due to their high risk of rapid bleeding and fatal outcomes if left untreated. We present a case of a hemodynamically unstable patient with a significant clot burden, successfully managed with a novel approach that revealed an LGAP with a penetrating gastric ulcer at the pseudoaneurysm site.

## 1 Introduction

Although gastric ulcers are a common cause of upper gastrointestinal bleeding (UGIB), the presence of a LGAP is a rare and potentially life‐threatening phenomenon. The reported incidence of pseudoaneurysms in UGIB is low, and LGAPs represent a small fraction of these cases. When present, however, they pose a diagnostic challenge. For example, clot burden can hinder the identification of bleeding sources, especially in hemodynamically unstable patients at risk for rapid deterioration. Therefore, effective clot management is crucial for visualization and diagnosis. Endoscopic tools, such as suction caps (e.g., distal caps) and prokinetic agents (e.g., erythromycin and metoclopramide), can manage clot burden, though their effectiveness varies [1]. Recent reports also suggest the potential use of an overtube connected with suction to suction out large clots from the stomach [2]. Our case illustrates a challenging scenario involving a large clot burden that interfered with visualization, requiring endoscopic tools for clot removal, ultimately revealing a rare left gastric artery pseudoaneurysm (LGAP) with an associated ulcer.

### 1.1 Case Report

**FIGURE 1:**
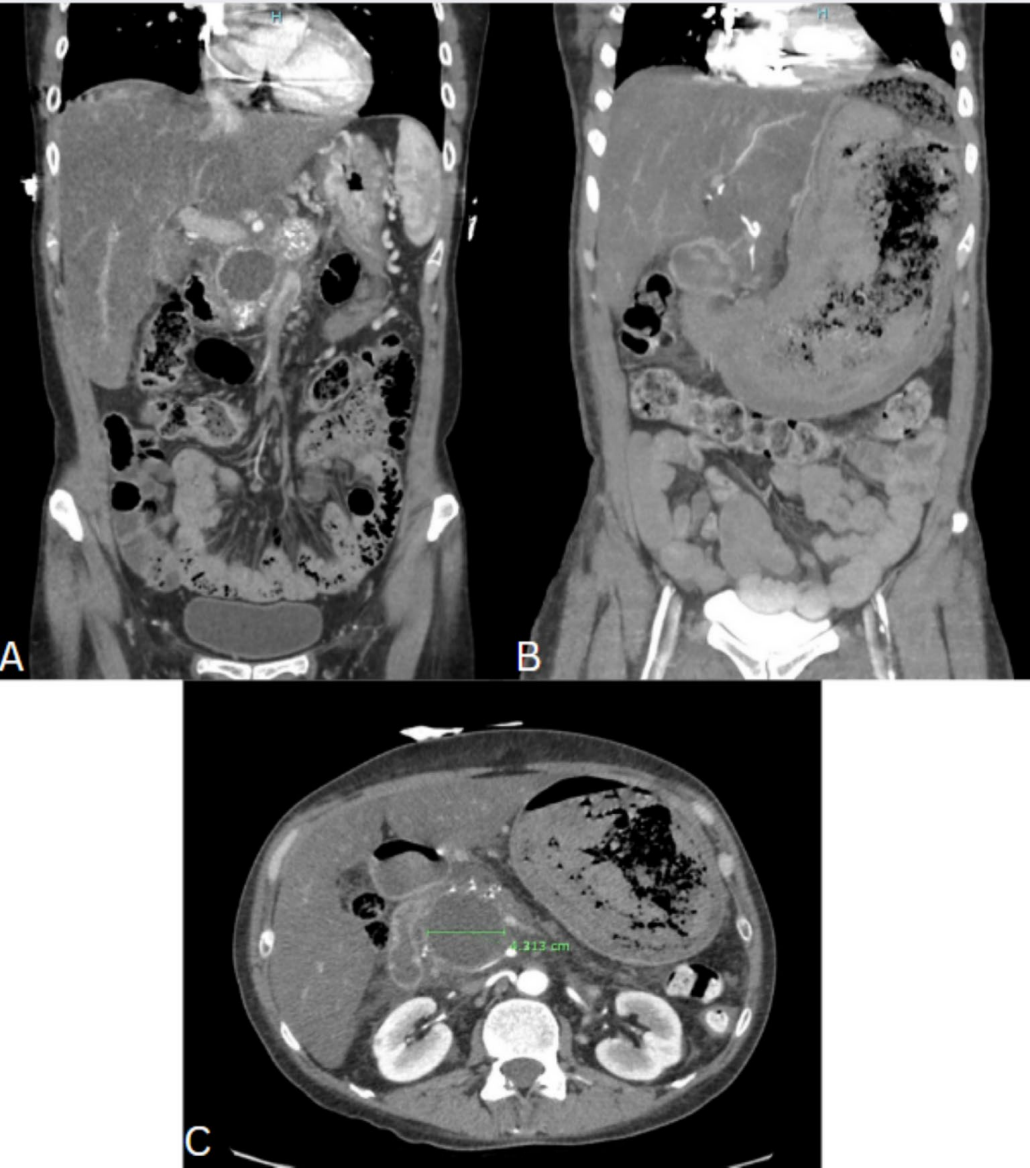
Initial CTA (coronal view) on admission for acute pancreatitis, demonstrating pseudocyst (A), followed by large amount of debris noted in the stomach on repeat CTA (coronal view) when transferred to the ICU on day 8 (B), and finally a 4.313 cm measured pseudocyst in the pancreatic head (C).

A 43‐year‐old female with deep vein thrombosis (DVT) on Apixaban, an automatic implantable cardiac defibrillator (AICD) for ventricular tachycardia and long‐QT syndrome, hepatic steatosis, and chronic pancreatitis complicated by chronic splenic vein thrombosis presented for recurrent alcoholic acute on chronic pancreatitis. She was managed on the general floor. She improved tremendously on IV fluids and pain medications and was nearing discharge. However, on day 8 of admission, she was transferred to the intensive care unit (ICU) for abrupt hematemesis requiring vasopressor support, with hemoglobin (Hgb) dropping from 11.1 to 8.6 g/dL. Her home daily proton pump inhibitor (PPI) was then doubled to twice a day and given through IV. Computed tomography angiography (CTA) suggested a pseudocyst (which was already known, increased in size from 3.6 to 4.3 cm and a distended gastric lumen filled with debris) (Figure 1), with no pseudoaneurysm nor active sites of bleeding seen. A nasogastric tube (NGT) was placed that retrieved maroon blood.

**FIGURE 2:**
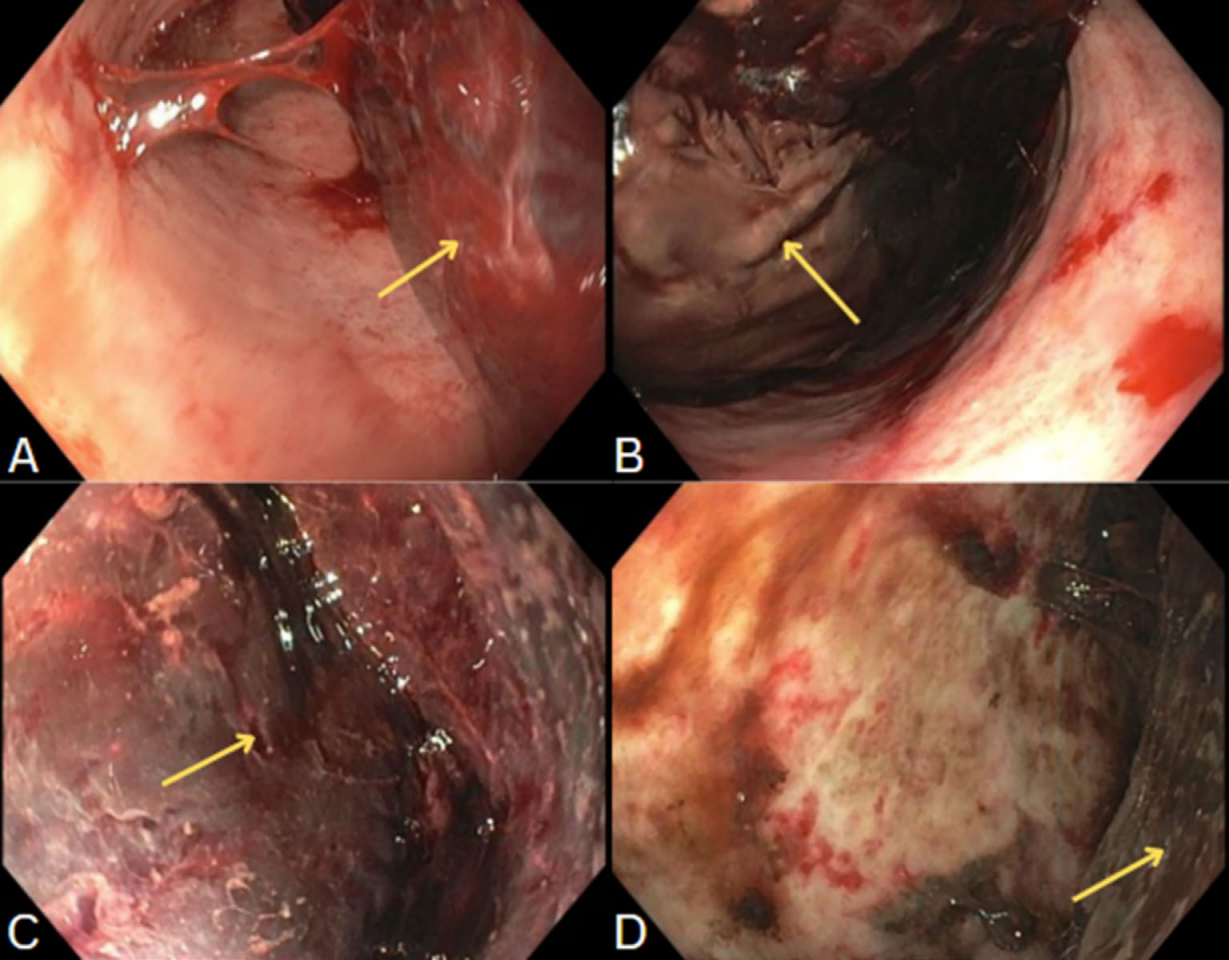
Gastric antrum demonstrating large clot burden (A), and gastric body redemonstrating clot (B), both from first endoscope. Large clot burden remains at the time of second look, even after metoclopramide for 24 h, as evidenced by gastric body photos (C, D).

An esophagogastroduodenoscopy (EGD) performed to evaluate for bleeding sources such as gastric varices or ulcers instead initially revealed a significant clot burden in the stomach, obscuring visualization of a bleeding source (Figure 2). Therefore, to assist with visualization, several doses of metoclopramide were administered, with serial electrocardiogram (EKG) monitoring. Her Hgb dropped to 5.3 g/dL overnight, prompting an emergent consultation with vascular and interventional radiology (VIR). A LGAP was identified and embolized. The patient's hemoglobin continued to drop slightly, encouraging a second EGD the following day, which still showed a large clot burden and obstructed view of any bleeding sources (Figure 2). Therefore, metoclopramide therapy was continued, and hemoglobin continued to be trended.

**FIGURE 3:**
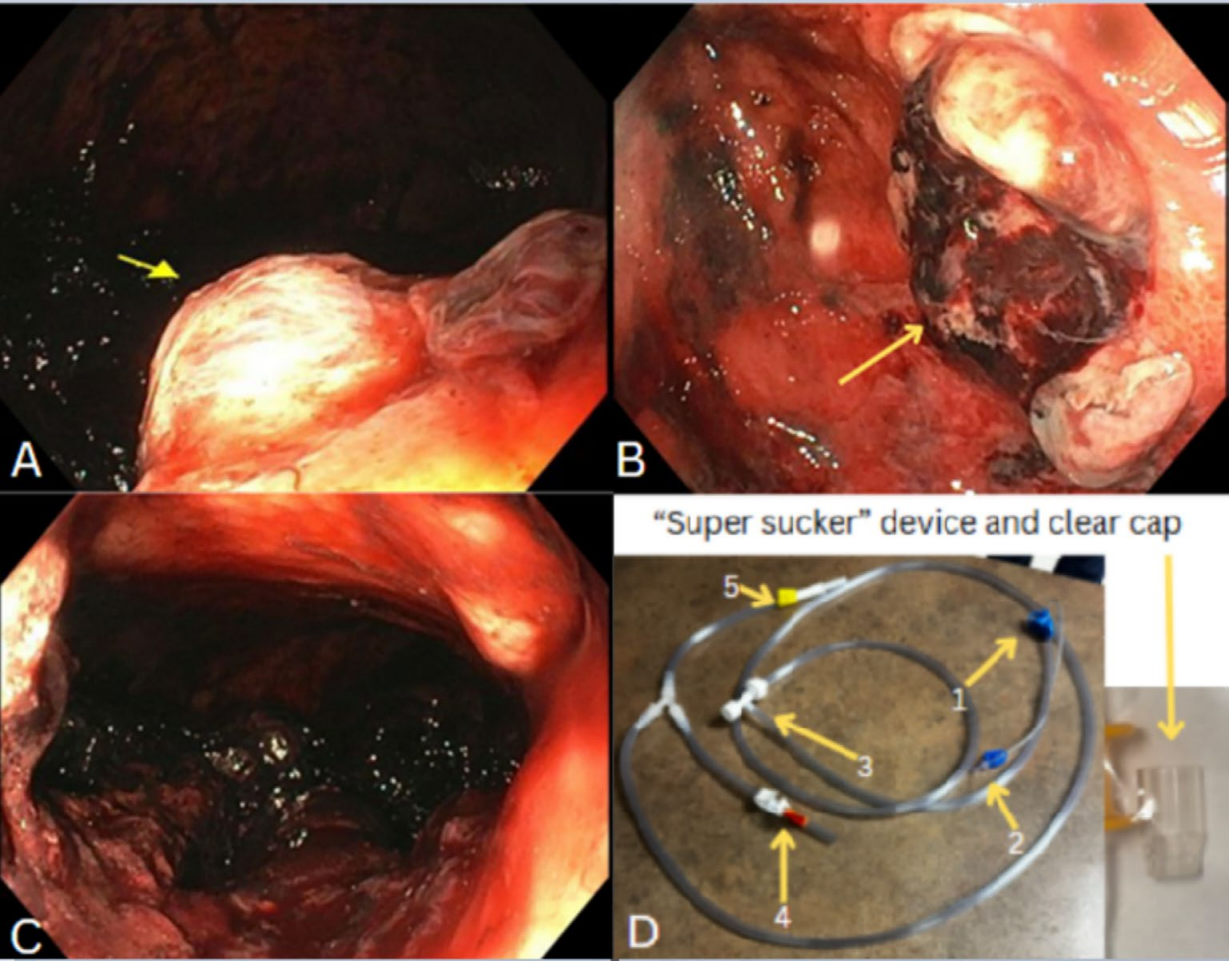
Aerial view of the lesser curvature ulcerated pseudoaneurysm (A), followed by a close‐up view of the same ulcerated pseudoaneurysm (B). Gastric body after 3 L of clots were suctioned and removed (C). Direct suction device with biopsy valve (1), irrigation line (2), trumpet valve (3), endoscopic suction (4), suction connector to suction canister (4), and clear cap on the right (D).

On the third EGD, the next day, a persistently high clot burden was seen. Therefore, a direct suction device (DSD) [3] was used to suction the clot with drastic improvement in clot burden. Residual clots were removed using a combination of the DSD and clear cap (CC) [4]. To remove the large clot burden, the DSD's trumpet valve (Figure 3) was methodically pressed and released until part of the clot was suctioned into the suction canister. The remaining clot in the stomach, due to the suction pressure of the DSD, fragmented into smaller pieces. These were then suctioned into the CC. Maintaining firm pressure and suction, the endoscope was then pulled out of the upper GI tract and the clot manually extracted from the CC. This was repeated many times until a total of 3 L of clots were removed, revealing a cratered ulcerated lesion in the proximal gastric body in the location of the left gastric artery without a visible vessel present nor bleeding (Figure 3). This anatomy corresponded with the embolized pseudoaneurysm. A hemostatic agent was then applied throughout the site.

By the time of transfer to the general floor, she had 1.2 L of blood suctioned through NGT, and 3 L of blood and clots removed endoscopically. The patient remained hemodynamically stable without any concerns for active bleeding and was eventually discharged after 16 days of hospitalization. She had a total of 11 units (u) of packed red blood cells (pRBC), 5u cryoprecipitate, 4u fresh frozen plasma (FFP), and 1u platelets given prior to medical stabilization. Follow‐up 1 month later revealed no recurrence of a gastric pseudoaneurysm or ulcer.

## 2 Discussion

Pancreatitis is often complicated by conditions such as pseudocysts, gastric outlet obstructions, and splenic vein thrombosis. Visceral pseudoaneurysms are not uncommon in patients with acute‐on‐chronic pancreatitis, although these tend to be rarer (around 10% in those with chronic pancreatitis). Given their high bleeding risk, these patients often present in hemorrhagic shock [5].

This case is unique and compelling for two reasons. First, LGAPs are extremely rare, with only a few case reports existing in the literature, and only three have described a penetrating gastric ulcer associated with the pseudoaneurysm [6]. When associated with both, the inherent risk of life‐threatening hemorrhage increases substantially, with some reports stating a mortality rate of 40%–80% [6]. Of all pseudoaneurysms related to pancreatitis, LGAP accounts for around 4% [7]; however, no current data exist on the incidence of gastric ulcers associated with LGAP.

The second reason is the adaptable and novel approach to managing the patient's substantial clot burden. Endoscopic tools such as large‐diameter suction devices and water irrigation with dilution are typically used to retrieve foreign objects or suction small quantities of fluids. In cases involving massive hemorrhage with difficult‐to‐assess pathology, techniques such as repositioning the patient or using external cannulas may aid in management [8, 9]. In this case, we used a combination of a DSD and a CC. While the CC is officially used for clip extraction and removal of ingested foreign objects, our practice and other practices have also used it for the management of food boluses; however, its use for clot removal remains unprecedented, to our knowledge [4, 10].

This case not only highlights the successful removal of the clot, revealing a rare ulcerated pseudoaneurysm, but is consistent with emerging evidence emphasizing the importance of creativity and real‐time problem‐solving when managing atypical, life‐threatening cases, especially when complicated by extensive clot burden. Such adaptability may pave the way for further exploration of endoscopic device applications. A high index of suspicion for vascular complications is crucial in severe UGIB, as demonstrated by the rare association of a LGAP and gastric ulcer in our case. Recognizing these complexities is crucial for timely hemostatic interventions in high‐risk patients.

## Consent

Informed consent was obtained for this case report.

## Conflicts of Interest

The authors declare no conflicts of interest.
